# Survival of Testicular Pure Embryonal Carcinoma vs. Mixed Germ Cell Tumor Patients across All Stages

**DOI:** 10.3390/medicina59030451

**Published:** 2023-02-24

**Authors:** Cristina Cano Garcia, Andrea Panunzio, Stefano Tappero, Mattia Luca Piccinelli, Francesco Barletta, Reha-Baris Incesu, Kyle W. Law, Lukas Scheipner, Zhe Tian, Fred Saad, Shahrokh F. Shariat, Derya Tilki, Alberto Briganti, Ottavio De Cobelli, Carlo Terrone, Alessandro Antonelli, Severine Banek, Luis A. Kluth, Felix K. H. Chun, Pierre I. Karakiewicz

**Affiliations:** 1 Department of Urology, University Hospital Frankfurt, Goethe University Frankfurt am Main, 60590 Frankfurt am Main, Germany; 2 Cancer Prognostics and Health Outcomes Unit, Division of Urology, University of Montréal Health Center, Montréal, QC H2X 0A9, Canada; 3 Department of Urology, University of Verona, Azienda Ospedaliera Universitaria Integrata di Verona, 37126 Verona, Italy; 4 Department of Urology, IRCCS Policlinico San Martino, 16132 Genova, Italy; 5 Department of Surgical and Diagnostic Integrated Sciences (DISC), University of Genova, 16132 Genova, Italy; 6 Department of Urology, IEO European Institute of Oncology, IRCCS, 20141 Milan, Italy; 7 Unit of Urology/Division of Oncology, Gianfranco Soldera Prostate Cancer Lab, IRCCS San Raffaele Scientific Institute, Vita-Salute San Raffaele University, 20132 Milan, Italy; 8 Martini-Klinik Prostate Cancer Center, University Hospital Hamburg-Eppendorf, 20246 Hamburg, Germany; 9 Department of Urology, Medical University of Graz, 8010 Graz, Austria; 10 Department of Urology, Comprehensive Cancer Center, Medical University of Vienna, 1090 Vienna, Austria; 11 Department of Urology, Weill Cornell Medical College, New York, NY 10065, USA; 12 Department of Urology, University of Texas Southwestern, Dallas, TX 75230, USA; 13 Hourani Center of Applied Scientific Research, Al-Ahliyya Amman University, Amman 19328, Jordan; 14 Department of Urology, University Hospital Hamburg-Eppendorf, 20246 Hamburg, Germany; 15 Department of Urology, Koc University Hospital, 34010 Istanbul, Turkey

**Keywords:** testicular cancer, pure embryonal, non-seminoma, cancer-specific mortality, SEER

## Abstract

*Background and Objectives*: The impact of pure histological subtypes in testicular non-seminoma germ cell tumors on survival, specifically regarding pure embryonal carcinoma, is not well established. Therefore, this study aimed to test for differences between pure embryonal carcinoma and mixed germ cell tumor patients within stages I, II and III in a large population-based database. *Materials and Methods*: We relied on the Surveillance, Epidemiology and End Results (SEER) database (2004–2019) to identify testicular pure embryonal carcinoma vs. mixed germ cell tumor patients. Cumulative incidence plots depicted cancer-specific mortality that represented the main endpoint of interest. Multivariable competing risks regression models tested for differences between pure embryonal carcinoma and mixed germ cell tumor patients in analyses addressing cancer-specific mortality and adjusted for other-cause mortality. *Results*: Of 11,223 patients, 2473 (22%) had pure embryonal carcinoma. Pure embryonal carcinoma patients exhibited lower cancer-specific mortality relative to their mixed germ cell tumor counterparts for both stage III (13.9 vs. 19.4%; *p* < 0.01) and stage II (0.5 vs. 3.4%, *p* < 0.01), but not in stage I (0.9 vs. 1.6%, *p* = 0.1). In multivariable competing risks regression models, pure embryonal carcinoma exhibited more favorable cancer-specific mortality than mixed germ cell tumor in stage III (hazard ratio 0.71, *p* = 0.01) and stage II (hazard ratio 0.11, *p* < 0.01). *Conclusions*: Pure embryonal carcinoma exhibits a more favorable cancer-specific mortality profile relative to mixed germ cell tumor in stage II and III testicular cancers. Consequently, the presence of mixed germ cell tumor elements may be interpreted as a risk factor for cancer-specific survival.

## 1. Introduction

Testicular germ cell tumors represent the most common solid tumor in men between 20 and 30 years. In the last decades, the incidence rates of testicular germ cell tumors increased, especially in a developed countries [1]. The treatment and prognosis of testicular germ cell tumors is based on primary tumor (T-stage), reginal lymph node (N-stage), distant metastases (M-stage), serum tumor markers (S-stage), as well as the distinctions between seminoma and non-seminoma germ ell tumors [1,2]. The impact of histological subtypes within the non-seminoma germ cell tumor, specifically regarding pure embryonal carcinoma as a risk factor for recurrence in stage I patients, has been addressed in previous studies [3,4,5,6,7,8]. However, the effect of pure embryonal carcinoma vs. mixed germ cell tumor, especially in advanced testicular cancer (stages II and III), on survival is not well established. Two institutional studies examined this topic reporting better cancer-specific survival in pure embryonal carcinoma vs. mixed germ cell tumor patients. For example, Dowling et al., relied on 93 stage II and 41 stage III pure embryonal carcinoma patients, of whom all were treated with chemotherapy and post-chemotherapy retroperitoneal lymph node dissection (PC-RPLND) with a median follow-up of 5.9 years [9]. Similarly, Bilen et al., relied on 22 stage II and 19 stage III pure embryonal carcinoma patients without reporting the median follow-up duration [10]. Based on the paucity of data regarding this topic, we tested for survival differences between pure embryonal carcinoma and mixed germ cell tumor patients in a large population-based database across all stages. Based on the reported survival advantages of pure embryonal carcinoma in the two previous series, we hypothesized that pure embryonal carcinoma patients exhibit more favorable survival than mixed germ cell tumor patients when testing for cancer-specific mortality. To test this hypothesis, we relied on the Surveillance, Epidemiology and End Results (SEER) database (2004–2019).

## 2. Materials and Methods

### 2.1. Patients

The SEER database approximates the United States demographic composition and cancer incidence by collecting cancer incidence and survival data from population-based cancer registries. Specifically, United States death data are provided from the National Center of Health Statistics (NCHS) to the SEER database, including the cause of death [11,12]. Within the SEER database from 2004 to 2019, we selected patients ≥ 18 years old, who underwent orchiectomy, with histologically confirmed testicular cancer (International Classification of Disease for Oncology (ICD-O) site codes C62.1, C62.9) of pure embryonal carcinoma (ICD-O-3 histology code 9070/3) or mixed germ cell tumor histology (ICD-O-3 Codes 9085/3). Autopsy only, as well as death certificate only cases as reporting sources were excluded. Further exclusion criteria consisted of unknown clinical stage and missing follow-up and survival data.

### 2.2. Statistical Analyses

Descriptive statistics included frequencies and proportions for categorical variables. Medians and interquartile ranges were reported for continuously coded variables. Wilcoxon rank sum test, Pearson Chi-square, and Fisher’s exact test tested for statistically significant differences in medians and proportions. All analyses stratified the population between pure embryonal carcinoma vs. mixed germ cell tumor. Separate models were first applied to stage I patients, then to stage II, and finally to stage III patients. Cumulative incidence plots depicted cancer-specific mortality rates in pure embryonal carcinoma vs. mixed germ cell tumor patients. Multivariable competing risks regression models tested for the independent predictor status of pure embryonal carcinoma vs. mixed germ cell tumor in analyses addressing cancer-specific mortality after adjustment for other-cause mortality, as well as in analyses addressing other-cause mortality after adjustment for cancer-specific mortality. Covariables consisted of age and RPLND status, as well as International Germ Cell Cancer Collaborative Group (IGCCCG) prognosis group and presence of lung metastases in stage III. In all statistical analyses, R software environment for statistical computing and graphics (R version 4.1.3, R Foundation for Statical Computing, Vienna, Austria) was used [13]. All tests were two-sided with a significance level set at *p* < 0.05. Owing to the anonymously coded design of the SEER database, study-specific ethics approval was waived by the institutional review board.

## 3. Results

### 3.1. Descriptive Characteristics

Of 11,223 study patients, 2473 (22%) had pure embryonal carcinoma vs. mixed germ cell tumor in 8750 (78%). Median age was 28 years in both groups. Median overall follow-up was 74 months (interquartile range: 29–127). Specifically, median follow-up was 74 months (interquartile range: 29–127) in stage I, 80 months (interquartile range: 34–125) in stage II, and 56 months (interquartile range: 17–118) in stage III. Pure embryonal carcinoma differed from mixed germ cell tumor patients. Pure embryonal carcinoma exhibited a higher clinical stage (stage I: 57 vs. 67%; stage II: 21 vs. 14%, stage III: 22 vs. 19%, *p* < 0.01) and higher rate of lung metastases (50 vs. 43%, *p* < 0.01). Conversely, pure embryonal carcinoma patients exhibited a lower rate of IGCCCG poor prognosis group than mixed germ cell tumor (30 vs. 39%; *p* < 0.01, Table 1).

### 3.2. Survival in Pure Embryonal Carcinoma and Mixed Germ Cell Tumor Patients

**Figure 1:**
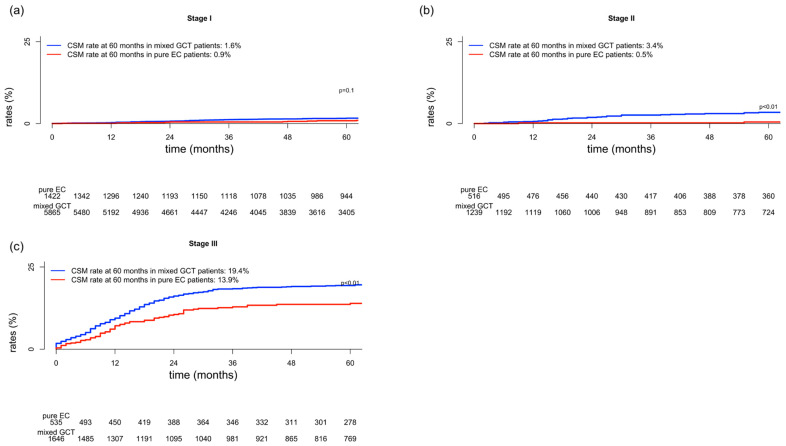
Cumulative incidence plots depicting cancer-specific mortality (CSM) in patients with pure embryonal carcinoma (EC) and mixed germ cell tumor (GCT) according to stage (**a**) I, (**b**) II, and (**c**) III.

Differences in cancer-specific mortality between pure embryonal carcinoma and mixed germ cell tumor in a stage-specific fashion were as follows: stage I 0.9 vs. 1.6% (*p* = 0.1; Figure 1a); stage II 0.5 vs. 3.4% (*p* < 0.01; Figure 1b); and stage III 13.9 vs. 19.4% (*p* = 0.03; Figure 1c). Differences in other-cause mortality between pure embryonal carcinoma and mixed germ cell tumor in a stage-specific fashion were as follows: stage I 1.2 vs. 1.2%; stage II 2 vs. 0.7%; and stage III 3.6 vs. 2.9%.

### 3.3. Multivariable Competing Risks Regression Models

Pure embryonal carcinoma was an independent predictor of lower cancer-specific mortality relative to mixed germ cell tumor in multivariable competing risks regression models that relied on stage II patients (hazard ratio: 0.11, 95% confidence interval: 0.03–0.43, *p* < 0.01), as well as in multivariable competing risks regression models that relied on stage III patients (hazard ratio 0.71, 95% confidence interval 0.55–0.93, *p* = 0.01). Conversely, in stage I, pure embryonal carcinoma did not differ from mixed germ cell tumor regarding cancer-specific mortality (hazard ratio 0.62, 95% confidence interval 0.82–6.07, *p* = 0.1).

Finally, no other-cause mortality differences in multivariable competing risks regression models were observed when pure embryonal carcinoma was compared to mixed germ cell tumor across all stages (stage I: hazard ratio 0.98, 95% confidence interval 0.65–1.47, *p* = 0.9; stage II hazard ratio 2.06, 95% confidence interval 0.98–4.35, *p* = 0.06, stage III: hazard ratio 1.23, 95% confidence interval 0.75–2.01, *p* = 0.4, Table 2).

## 4. Discussion

Survival data regarding pure embryonal carcinoma vs. mixed germ cell tumor patients, especially in advanced testicular cancer (stages II and III) patients, are limited. Based on the advantage of pure embryonal carcinoma reported in previous small-sized institutional analyses, we tested for cancer-specific mortality differences in a stage-specific fashion, between pure embryonal carcinoma and mixed germ cell tumor patients in a large population-based cohort. We hypothesized that similar survival patterns may be recorded according to previous smaller-scale historical reports. We tested this hypothesis within the 2004–2019 SEER database and made several important observations.

First, we observed important differences in stage distribution, presence of lung metastases, and IGCCCG prognosis group between pure embryonal carcinoma vs. mixed germ cell tumor patients. Specifically, pure embryonal carcinoma exhibited higher rates of stage II (21 vs. 14%) and stage III (22 vs. 19%) than mixed germ cell tumor patients and lower rates of stage I (57 vs. 67%; *p* < 0.01). These differences are in agreement with Bilen et al., where pure embryonal carcinoma patients exhibited higher rates of stage II and III relative to mixed EYT (embryonal, yolk sac, and teratoma), as well as relative to mixed EYTS (embryonal, yolk sac, teratoma, and seminoma) patients [10]. Conversely, IGCCCG poor prognosis was less frequent in pure embryonal carcinoma patients than in mixed germ cell tumor patients in the current study (49 vs. 39%; *p* < 0.01). These differences agree with Dowling et al., where IGCCCG poor prognosis membership was lower in pure embryonal carcinoma patients than in PC-RPLND treated mixed germ cell tumor patients [9]. Finally, lung metastases were more frequent in pure embryonal carcinoma than in mixed germ cell tumor in the current study (50 vs. 43%; *p* < 0.01). To the best of our knowledge, we are the first to report these differences in lung metastases rates between pure embryonal carcinoma vs. mixed germ cell tumor patients. Consequently, this finding cannot be directly compared with any previous work. Taken together, certain characteristics of pure embryonal carcinoma relative to mixed germ cell tumor appear more favorable, but other characteristics appear less favorable regarding cancer control. Therefore, the presence of pure embryonal carcinoma at baseline cannot be unequivocally considered a cancer control disadvantage.

Second, we recorded important differences in cancer-specific mortality between pure embryonal carcinoma and mixed germ cell tumor after accounting for the potential confounding effect of other-cause mortality using competing risks regression modeling. Specifically, in stages II and III in pure embryonal carcinoma we recorded lower cancer-specific mortality rates than that of mixed germ cell tumor (stage II: 0.5 vs. 3.4%, *p* > 0.01; multivariable hazard ratio 0.11, *p* < 0.01; stage III: 13.9 vs. 19.4%, *p* < 0.01; multivariable hazard ratio 0.71, *p* = 0.01). Our findings are in agreement with Bilen et al., comparing the survival of 68 pure embryonal carcinoma patients vs. 207 mixed germ cell tumor patients [10]. In their series, pure embryonal carcinoma patients of all stages exhibited a particularly favorable treated natural history since no events in cancer-specific mortality were recorded within the subgroup of 68 pure embryonal carcinoma patients. Of those, 19 (28%) were stage III, 22 (32%) were stage II, and 27 (40%) were stage I pure embryonal carcinoma patients. Absence of events in cancer-specific mortality for stage III patients that were included in the comparison strongly suggests good prognosis profile. However, this interpretation cannot be validated based on objective data from Bilen et al., that were not included in the report, since neither IGCCCG prognosis groups nor median follow-up duration were provided [10]. Consequently, it may be possible that the very favorable profile according to cancer-specific mortality of these 19 stage III pure embryonal carcinoma patients might be related to a very short follow-up and limited data maturity. Similarly, Dowling et al., also provided combined outcomes in cancer-specific mortality of 145 pure embryonal carcinoma vs. 960 mixed germ cell tumor patients treated with chemotherapy and PC-RPLND without stage stratification and without IGCCCG prognosis group stratification in stage III patients [9]. Their results illustrate a five-year cancer-specific mortality rate of 1.7% in pure embryonal carcinoma patients, of whom the majority were stage II patients (93, 64%), followed by 41 (28%) stage III patients, and 11 (8%) stage I patients. Consequently, in the study of Dowling et al., stage III patients represented less than one third of the overall cohort [9]. Moreover, IGCCCG poor and intermediate prognosis group patients, in whom cancer-specific mortality is more elevated, accounted for marginal proportions of 3.4 and 4.1%, respectively. Regardless of the underlining explanation for the findings reported by Dowling et al. and Bilen et al., the current results that are based on cancer-specific mortality rates of 13.9% in stage III, 0.5% in stage II, and 0.9% indicate cancer-specific mortality rates that are clearly higher in pure embryonal carcinoma for stage III, but comparable in pure embryonal carcinoma for stage II and I vs. mixed germ cell tumor [9,10].

The clinical implications of our observations are as follows. First, pure embryonal carcinoma is a rare entity, and the natural history of pure embryonal carcinoma is difficult to study and compare to mixed histology unless very large-scale epidemiological databases are available. In consequence, data from tertiary care centers with high volumes of testicular cancer are valuable. However, they only provide small samples that are heavily weighted towards stage II or favorable prognosis stage III patients according to Dowling et al. and Bilen et al. [9,10]. In stage II and favorable prognosis stage III patients, cancer-specific mortality rates after treatment are very low, and clinically meaningful differences are difficult to identify. In that regard, the SEER database provides a unique opportunity for identifying a very large number of pure embryonal carcinoma testicular cancer patients that is 17 times larger than that described by Dowling et al., and 36 times larger than that described by Bilen et al. [9,10]. Second, this substantially larger patient cohort allows better comparisons that are not undermined by very limited or even nonexistent cancer-specific mortality rates. Moreover, stage-specific comparisons could be made. Additionally, in stage III patients, adjustment for IGCCCG prognosis group was also included in multivariable comparisons between pure embryonal carcinoma and mixed germ cell tumor. Finally, all cancer-specific mortality comparisons were also adjusted for other-cause mortality. This potential confounder of other-cause mortality was not adjusted by Dowling et al. [9]. All methodological steps demonstrated and validated more favorable cancer-specific mortality in pure embryonal carcinoma patients. Conversely, our data corroborated data by Dowling et al. and Bilen et al., regarding the unfavorable effect of mixed germ cell tumor on cancer-specific mortality [9,10]. In consequence, advanced stage (stages II and III) testicular cancer patients harboring mixed germ cell tumor should be strongly considered at risk of worse outcomes when mortality represents the endpoint of interest.

Despite its novelty, the current study has several limitations. The first and foremost limitation consists of patient origin. Specifically, our findings are applicable to individuals who are identified within the SEER database. Consequently, the observations made within the current study are not generalizable to individuals from outside the United States or even patients who are not comparable to those included in the SEER database. For example, heterogeneity may be assumed according to the expertise and management of testicular cancer patients since rates of patients treated at cancer centers of excellence is unknown within the SEER database. Second, we relied on a large-scale retrospective series. However, the two existing reports that addressed this topic were also retrospective in nature and originated from single institutional databases [9,10]. Consequently, the current retrospective series has certain characteristics that represent an advantage to previous retrospective series. Sample size and larger proportion of stage III patients of all IGCCCG prognosis groups represent two main advantages. Conversely, other characteristics, such as detailed cancer information, are clearly less complete than those of the two institutional reports. Third, the median follow-up of 6 years (74 months) represents another limitation of the current study. Ideally longer follow-up would be of value. However, the current study follow-up compares favorably with Dowling et al., (median follow-up of 5.9 years) [9]. Median follow-up duration was not reported by Bilen et al. [10]. Consequently, it cannot be directly compared with the current study. Fourth, the stage distribution of the current study is heavily weighted in favor of stage I (57% of pure embryonal carcinoma patients). In the series by Dowling et al., 64% of pure embryonal carcinoma patients represented stage II vs. 21% in the current study (9). However, this proportion was based on only 39 observations vs. 516 observations in the current study. Similarly, Dowling et al., included a larger proportion of stage III pure embryonal carcinoma patients of 28% vs. 22% in the current study. However, this proportion only reflected 41 patients vs. 535 in the current study. Therefore, despite lower percentages of stage II and III, the current series provided a substantially larger number of observations to draw conclusions about cancer-specific mortality. Fifth, our series provided stage-specific cancer-specific mortality rates but not IGCCCG prognostic-group-specific rates in stage III patients. This limitation was shared with Dowling et al. and Bilen et al. [9,10]. However, these two previous reports did not provide IGCCCG prognostics-group-specific or stage-specific cancer-specific mortality rates. Consequently, the current series provides more detailed outcomes that were previously unknown. Moreover, the current study illustrates the presence of pronounced differences in cancer-specific mortality between stage III (13.9%) vs. stage II (0.5%) vs. stage I (0.9%). Sixth, we provided mortality rates. However, the nature of the SEER database did not allow us to address early cancer control outcomes, such as relapse rates or metastatic progression rates. These endpoints were addressed in the two previous series. Consequently, all available retrospective series provide complementary results that add to the existing knowledge base regarding pure embryonal carcinoma vs. mixed germ cell tumor. Seventh, no information about centralized pathological review was available within the SEER database. Finally, the SEER database lacks specific histological information about the composition of mixed germ cell tumor and the percentages of various components within the primary.

## 5. Conclusions

In conclusion, pure embryonal carcinoma exhibits a more favorable cancer-specific mortality profile relative to mixed germ cell tumor in stage II and III testicular cancers. Consequently, the presence of mixed germ cell tumor elements may be interpreted as a risk factor for cancer-specific survival.

## Figures and Tables

**Table 1:** Baseline characteristics of 11,223 patients with pure embryonal carcinoma (EC) and mixed germ cell tumor (GCT) within the SEER (2004–2019).

Characteristic	Pure EC ^1^ *n* = 2473 (22%)	Mixed GCT ^1^ *n* = 8750 (78%)	*p*-Value ^2^
Age at diagnosis (years)	28 (24, 34)	28 (24, 35)	0.2
Stage			<0.01
I	1422 (57%)	5926 (67%)	
II	516 (21%)	1249 (14%)	
III	535 (22%)	1666 (19%)	
RPLND performed	460 (19%)	1636 (19%)	0.9
Serum tumor markers (S-stage)			<0.001
S0	507 (21%)	1563 (18%)	
S1	54 (2%)	268 (3%)	
S2	186 (8%)	804 (9%)	
S3	58 (2%)	394 (5%)	
S0	1668 (67%)	5721 (65%)	
IGCCCG prognosis group for stage III (*n* = 2181)	*n* = 535	*n* = 1646	<0.01
Good prognosis	104 (19%)	271 (16%)	
Intermediate prognosis	97 (18%)	369 (22%)	
Poor prognosis	160 (30%)	639 (39%)	
Unknown	174 (33%)	367 (22%)	
Presence of lung metastases	269 (50%)	713 (43%)	<0.01

^1^ Median (interquartile range); *n* (%), ^2^ Wilcoxon rank sum test; Pearson’s Chi-square test; Fisher’s exact test.

**Table 2:** Multivariable competing risks regression models predicting cancer-specific mortality (CSM) and other-cause mortality (OCM) in patients with pure embryonal carcinoma (EC) or mixed germ cell tumor (GCT) in stages I and II (adjusted for age and RPLND), as well as stage III (adjusted for age, RPLND, IGCCCG prognosis group, and presence of lung metastases).

		CSM		OCM	
		Multivariable HR (95% CI)	*p*-Value	Multivariable HR (95% CI)	*p*-Value
Stage I					
Histology	Mixed GCT	Reference	-	Reference	-
	Pure EC	0.62 ^a^ (0.35–1.09)	0.1	0.98 ^a^ (0.65–1.47)	0.92
Stage II					
Histology	Mixed GCT	Reference	-	Reference	-
	Pure EC	0.11 ^a^ (0.03–0.43)	<0.01	2.06 ^a^ (0.98–4.35)	0.06
Stage III					
Histology	Mixed GCT	Reference	-	Reference	-
	Pure EC	0.71 ^b^ (0.55–0.93)	0.01	1.23 ^b^ (0.75–2.01)	0.42

^a^ adjusted for age and RPLND; ^b^ age, RPLND, IGCCCG prognosis group and presence of lung metastases. Abbreviations: HR = hazard ratio, CSM = cancer-specific mortality, OCM = other-cause mortality, GCT = germ cell tumor, EC = embryonal carcinoma, RPLND = retroperitoneal lymph node dissection, IGCCG= International Germ Cell Cancer Collaborative Group.

## Data Availability

The data presented in this study are available on request from the corresponding author.
